# Influence of different playing styles among the top three teams on action zones in the World Cup in 2018 using a Markov state transition matrix

**DOI:** 10.3389/fpsyg.2022.1038733

**Published:** 2022-11-18

**Authors:** Tianbiao Liu, Chenye Zhou, Xumei Shuai, Li Zhang, Jingjing Zhou, Lang Yang

**Affiliations:** ^1^College of Physical Education and Sports Science, Beijing Normal University, Beijing, China; ^2^College of Biological Sciences and Technology, Beijing Forestry University, Beijing, China; ^3^School of Statistics, Beijing Normal University, Beijing, China; ^4^Zhongguancun Foreign Language School, Beijing, China

**Keywords:** soccer, Markov chain, stochastic process, playing behaviors, playing style, offensive sequence, zone, performance analysis

## Abstract

**Purpose:**

In football, attacking has seen evolving for decades and attacking pattern detection is an important topic in this sport. The purpose of this study was to identify the general and threatening attacking patterns of different playing styles in world top football matches, which represented the latest evolvement of soccer attacking.

**Methods:**

Attacking sequence data of the top three teams from 21 matches in the 2018 World Cup were collected. The three teams were classified into two playing styles according to a previous study, France was a direct-play team, and Croatia and Belgium were possession-play teams. The football field was divided into 12 zones and Markov transition matrix-based zone models were applied to assess the attacking pattern in the 21 matches. Both descriptive analysis and simulative analysis were conducted using this model.

**Results:**

The results revealed that (1) flanker attacks were frequently taken among all three teams, and possession playing teams (Croatia and Belgium) played more often than direct playing teams (France) in their center of the midfield zone and (2) forward passes across/through zones toward the middle of attacking quarter (A1/4) have a positive impact of creating a chance of a goal.

**Conclusion:**

Using Markov transition matrix, general and threatening attacking patterns were found. The combination of possession play and counterattack was a new trend that emerged in the 2018 World Cup. These findings can help coaches to develop corresponding strategies when facing opponents of different playing styles.

## Introduction

For team sports such as football, the overall strength of the team is not equal to the linear accumulation of the athletic abilities of all of the players on the pitch. The team’s performance is influenced by many factors; thus, the traditional descriptive statistical method can neither provide global information about the game (Glazier, 2010) nor convert a single variable into tactical information (Pfeiffer and Perl, 2006). Therefore, some studies have used nonlinear models to evaluate the performance of collective events (such as ball games). Lames (2006) believes that football matches are complex systems. Therefore, the concept of the relative phase is introduced into the game analysis to analyze football matches by establishing a nonlinear model (Lames, 2006). Afterward, decision tree technology (Lin, 2011), the Apriori algorithm (Pan, 2010; Liu and Hohmann, 2013a) and its improved version (Tianbiao and Andreas, 2016), and the sequential pattern mining algorithm (Liu et al., 2018) are used to analyze the set-piece tactics and attack patterns in football matches. Moreover, by applying social network analysis methods, Cao et al. (2019) analyzed the group combination of players in the football game, and Wu et al. (2020) and Yu et al. (2020) analyzed the importance of player positions in the match and the performance of foreign players. These studies analyzed the relationship between players by constructing a passing network and used network parameters to evaluate the performance of the players. The lines connecting the nodes in these passing networks represent passing activities.

Passing is one of the most important behaviors in the football field. The team organizes the offense by passing the ball, thus creating scoring opportunities. Current research on passing has mainly focused on counting the number of passes in the game and evaluating the relationship between various passes and winning (Shafizadeh et al., 2013; Liu et al., 2016, 2019). However, simple quantitative research cannot describe complex passing behaviors (Rein et al., 2017), nor can it support the research of passing decision-making. In particular, it is difficult to help coaches and players to apply any behavioral data in practice (Rein et al., 2017). Due to the characteristics of using technical and tactical behaviors in different areas of the football ground, as well as the different pressures given by opponents (Chen et al., 2015), it is particularly important to add position and field information when studying passing, technical, and tactical indicators (Fournier-Viger et al., 2019; Li and Zhang, 2019).

For each team, the passing and the use of the court area have their own characteristics. Camerino et al. (2012) studied the playing style of FC Barcelona and identified two different attacking patterns (T-Patterns), which contained different areas and passing routines. Moreover, Yi et al. (2019) observed that the technical and tactical performance and running of teams of different playing styles are different; in addition, by using technical and tactical indicators, it is possible to distinguish teams of different game styles (Lago-Peñas et al., 2017). Traditionally, it is believed that there are two typical playing styles (possession play and direct play; Hughes and Franks, 2005; Kempe et al., 2014) during offending. Later, combining with defending styles, researchers developed this playing style theory and more styles were identified (Fernandez-Navarro et al., 2016; Lago-Peñas et al., 2017; Castellano and Pic, 2019). The difference in the playing style is related to culture, football philosophy, and the skill levels of players. Ball possession is one of the most commonly used indicators to distinguish these two styles of play (Hewitt et al., 2016), which also influences the technical and physical performances of teams and players (Bradley et al., 2013; Liu et al., 2021). Additionally, situational variables also play an important role and affect playing styles (Fernandez-Navarro et al., 2018).

As a vital algorithm for the mathematical simulation of performance diagnoses, the Markov chain model has been applied to diagnostic analysis of net sports, such as table tennis (Zhang, 2003; Pfeiffer et al., 2010; Wenninger and Lames, 2016), tennis (Lames, 1991), and volleyball (Miskin et al., 2010; Hileno et al., 2020). In invasion games, the Markov chain state transition matrix can be used to describe and diagnose important passes in football (Liu and Hohmann, 2013b; Liu, 2014) or important connections in frisbee (Lam et al., 2021). Although there have been other important pattern detection technologies in the research of football (Liu and Hohmann, 2013a; Sarmento et al., 2014b), such as T-pattern (Borrie et al., 2002; Camerino et al., 2012; Pic Aguilar, 2017), these studies only aimed to describe general attacking pattern and did not offer a simulative way to discover threatening patterns. Moreover, most of the previous studies in this field did not include different playing styles in the analysis (Hirotsu and Wright, 2002; Wright and Hirotsu, 2003). Therefore, this study considers the attack sequences of the top three teams in the 2018 World Cup (the champion France, the second place Croatian, and the third place Belgium teams) as the research objects and explores the offensive routes and covering areas of the world’s top national teams with different playing styles in the World Cup.

## Materials and methods

### Samples

As shown in Table 1, this study recorded a total of 13,666 passing events in 21 games (7 games per team) of the top three teams (France, Croatia, and Belgium) in the 2018 World Cup in Russia. The dataset contained the time, area, and player of each pass. In the 21 recorded games, there were 10 games against possession-play opponents and 11 games against direct-play opponents. According to the division of Yi et al. (2019), among the top three teams, France was a direct-play team, and Croatian and Belgian teams exhibited possession-play styles. Among the opponents in the competition, Denmark, Russia, Iceland, Nigeria, Panama, Tunisia, Uruguay, and Peru were direct-play teams, whereas England, Argentina, Brazil, Japan, and Australia were possession-play teams.

**Table 1:** General description of research samples.

Match ID	Date	Observed team	Opposing team	Playing style of opposing teams	Observed team passing numbers	Observed team possession	Opposing team possession	Match result	Phase
1	2018-07-07	Belgium	Brazil	Possession play	493	46.03%	**53.97%**	2–1	1/4 final
2	2018-07-11	Belgium	England	Possession play	587	**56.76%**	43.24%	2–0	3–4 final
3	2018-06-29	Belgium	England	Possession play	647	**50.72%**	49.28%	1–0	group stage
4	2018-06-16	Belgium	France	Direct play	709	**54.52%**	45.48%	0–1	semifinal
5	2018-07-11	Belgium	Japan	Possession play	723	**55.31%**	44.69%	3–2	1/8 final
6	2018-06-18	Belgium	Panama	Direct play	710	**56.85%**	43.15%	3–0	group stage
7	2018-06-23	Belgium	Tunisia	Direct play	542	**55.97%**	44.03%	5–2	group stage
8	2018-06-22	Croatia	Argentina	Possession play	564	48.05%	**51.95%**	3–0	group stage
9	2018-07-02	Croatia	Denmark	Direct play	650	**53.94%**	46.06%	4–3	1/8 final
10	2018-07-12	Croatia	England	Possession play	662	47.01%	**52.99%**	2–1	semifinal
11	2018-07-15	Croatia	France	Direct play	713	47.15%	**52.85%**	2–4	final
12	2018-06-24	Croatia	Iceland	Direct play	697	**62.61%**	37.39%	2–1	group stage
13	2018-06-27	Croatia	Nigeria	Direct play	650	**53.87%**	46.13%	2–0	group stage
14	2018-07-08	Croatia	Russia	Direct play	803	**51.26%**	48.74%	6–5 (2–2)	1/4 final
15	2018-06-30	France	Argentina	Possession play	513	38.63%	**61.37%**	4–3	1/8 final
16	2018-06-16	France	Australia	Possession play	728	**69.23%**	30.77%	2–1	group stage
17	2018-07-11	France	Belgium	Possession play	557	45.48%	**54.52%**	1–0	semifinal
18	2018-07-15	France	Croatia	Possession play	515	**52.85%**	47.15%	4–2	final
19	2018-06-26	France	Denmark	Direct play	866	**54.88%**	45.12%	0–0	group stage
20	2018-06-21	France	Peru	Direct play	595	**56.46%**	43.54%	1–0	group stage
21	2018-07-06	France	Uruguay	Direct play	742	**55.16%**	44.84%	2–0	1/4 final

Bold values mean higher ball possession in the match.

### Division of football field

**Figure 1:**
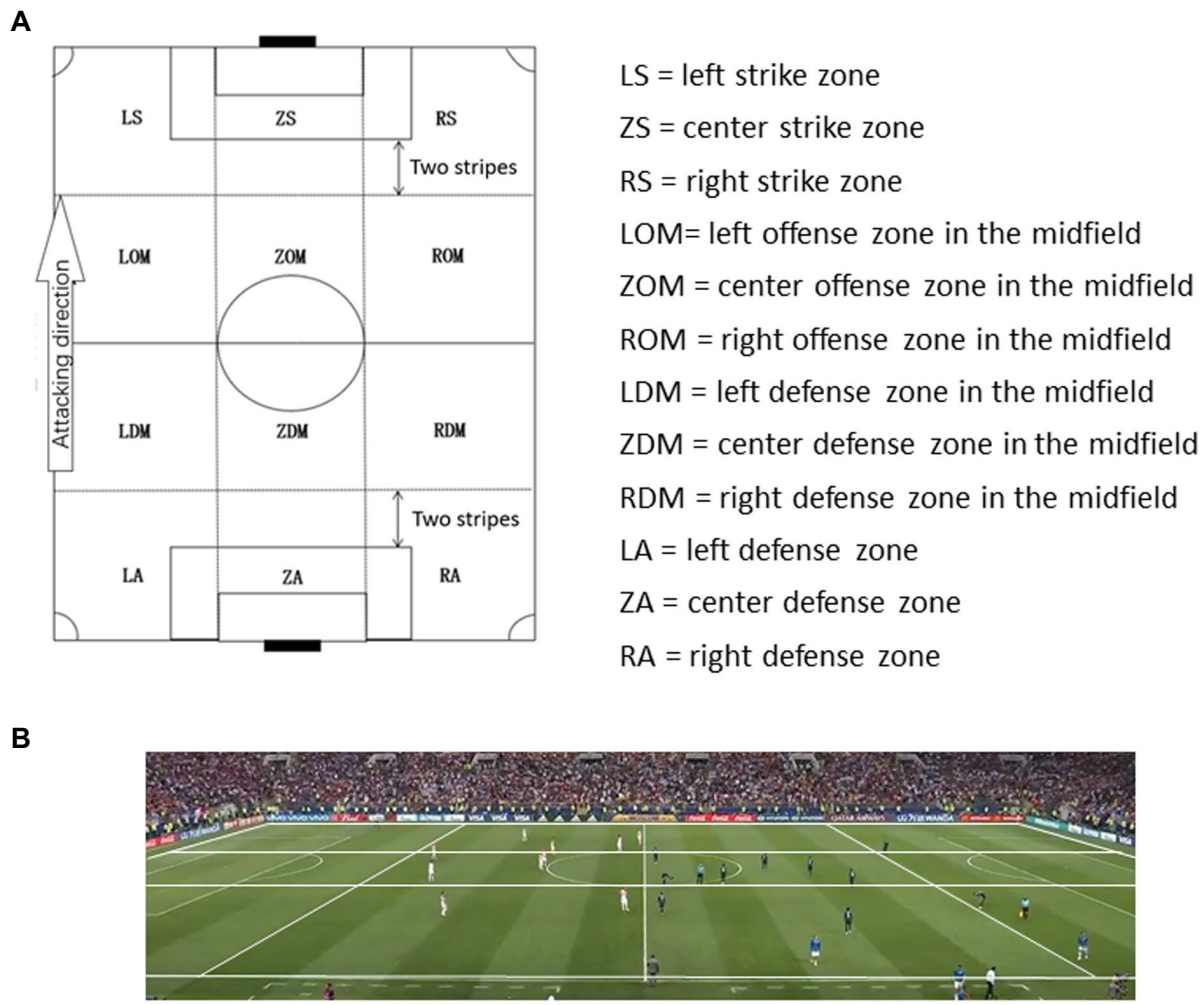
**(A)** Division of football ground. **(B)** Zone and turf band of football ground.

The World Cup football field is based on FIFA standards, with a length of 105 m and a width of 68 m. According to a previous study (Pfeiffer et al., 2006), the football field is divided into four fields according to the front field, center front field, center backfield, and backfield. Each field is divided into three areas: left, center, and right areas, with a total of 12 areas (Figure 1A). Moreover, the division of the field is divided according to the grass stripes. Each half of the World Cup venue has 10 horizontal grass stripes; therefore, each field section contains 5 horizontal grass stripes (Figure 1B).

### Data collection

All of the game videos were collected from the World Cup 2018 homepage of CCTV (China Central Television), which holds exclusive media rights to FIFA World Cup 2018. Two experienced observers observed the game video and recorded the passing information in the game according to the division of the field. To verify the reliability of the data, two matches were randomly selected; furthermore, a Cohen’s Kappa test was run for the two sets of data, and k = 0.61. According to Landis and Koch (1977) and Fleiss et al. (2013), the data have good consistency (Substantial) and can be used in research.

### Game observation model

**Figure 2:**
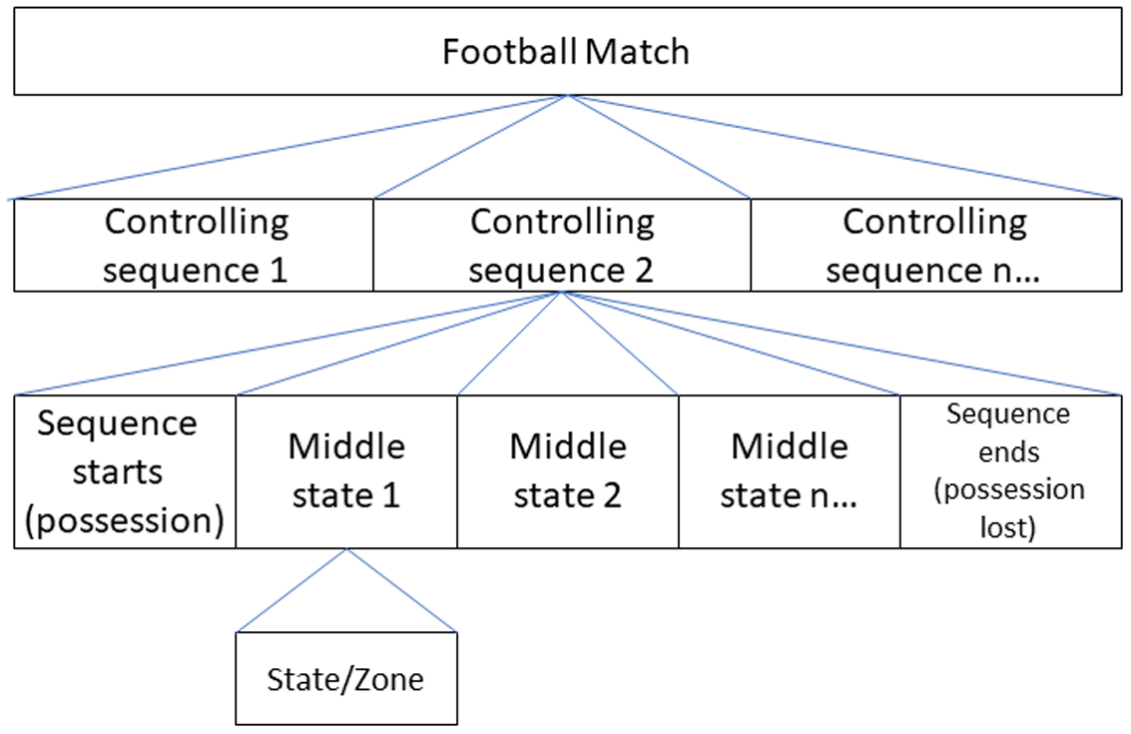
Sequence of ball possession and state in football.

Based on previous studies (Liu and Hohmann, 2013b; Liu, 2014), during the game, each area that the ball passes through is regarded as a state, the process consisting of passing is regarded as a ball control sequence (chain), and each ball control sequence (Chain) is composed of several intermediate states (zones). The sequence of possession (chain) is initiated with the possession of the ball (specifically, the starting state) and ends with the loss of possession (Figure 2; Liu and Hohmann, 2013b). The absorbing state is defined as whether a scoring opportunity is created when there is a loss of possession of the ball (TC = opportunity is created; NTC = opportunity is not created; Tianbiao and Andreas, 2016).

### Statistical model and data processing

#### Constructing the game state transition matrix

Passing between different zones is regarded as the transition between the game states. A two-dimensional state transition probability matrix can be constructed through the state transition probability. Each element in the matrix is a positive percentage number that is not greater than 100%, and the sum of any row elements in the matrix is 100%. Table 2 shows an example of state transition probability matrix from one match. Therefore, besides calculating general descriptive results of the passing paths, according to Lames (1991), the state transition probability matrix of the game can be used to calculate the probability of creating a scoring opportunity through a Markov chain (Liu and Hohmann, 2013b).

**Table 2:** Example of state transition probability matrix for zones.

To	LA	ZA	RA	LDM	ZDM	RDM	LOM	ZOM	ROM	LS	ZS	RS	NTC	TC
**From**														
LA	30.95%	4.76%	0.00%	30.95%	0.00%	0.00%	4.76%	2.38%	0.00%	0.00%	0.00%	2.38%	23.81%	0.00%
ZA	3.92%	11.76%	7.84%	0.00%	15.69%	5.88%	3.92%	19.61%	17.65%	0.00%	0.00%	1.96%	7.84%	3.92%
RA	0.00%	6.25%	37.50%	3.13%	0.00%	21.88%	0.00%	6.25%	3.13%	0.00%	0.00%	0.00%	21.88%	0.00%
LDM	4.36%	0.00%	0.00%	42.03%	8.70%	0.00%	18.84%	2.90%	0.00%	2.90%	1.45%	1.45%	17.39%	0.00%
ZDM	2.94%	5.88%	0.00%	5.88%	20.59%	11.76%	5.88%	2.94%	14.71%	0.00%	0.00%	5.88%	23.53%	0.00%
RDM	1.79%	1.79%	1.79%	5.36%	3.57%	41.07%	3.57%	1.79%	8.93%	0.00%	0.00%	5.36%	23.21%	1.79%
LOM	3.28%	0.00%	0.00%	4.92%	4.92%	1.64%	29.51%	1.64%	1.64%	11.48%	0.00%	3.28%	34.43%	3.28%
ZOM	0.00%	2.94%	0.00%	0.00%	2.94%	0.00%	11.76%	26.47%	8.82%	2.94%	0.00%	5.88%	38.24%	0.00%
ROM	0.00%	0.00%	0.00%	0.00%	1.60%	4.76%	0.00%	1.59%	42.86%	1.59%	0.00%	7.94%	36.51%	3.17%
LS	0.00%	0.00%	0.00%	7.14%	0.00%	0.00%	14.29%	0.00%	0.00%	7.14%	0.00%	0.00%	57.14%	14.29%
ZS	0.00%	0.00%	0.00%	0.00%	0.00%	0.00%	0.00%	0.00%	0.00%	0.00%	16.67%	8.33%	0.00%	75.00%
RS	0.00%	0.00%	0.00%	0.00%	0.00%	2.17%	0.00%	0.00%	0.00%	0.00%	0.00%	36.96%	41.30%	19.57%

#### Markov chain

In this study, zone was used as the research variable. In the game, players pass the ball from one zone to the next. This study regards the ball’s trajectory as a random system, and the zone where the ball is located at a certain moment is defined as a state.

Let $X_{n}$ be the state of the ball at time *n*; then, according to the Markov chain formula (Ching and Ng, 2006):


(1) $$P\left\{X_{n+1}=k|X_{n}=j\right\}=P_{jk}\left(j,k=1,2,\ldots ,n\right)$$

It can be expressed as follows: at the *n^th^* time, the ball is in zone *j*, and at the *(n + 1)^th^* time, the probability of the ball passing to zone *k* is the probability of the transition from state *j* to state *k*.

In the game, the state of the ball (the zone where the ball is located) at a certain time in the future is only related to the current state (zone) and has nothing to do with its previous state (zone), which is consistent with Markov chain’s “no aftereffect.” In addition, the time parameter of the possession chain is a discrete process, which can be represented by a sequence of $X_{n}$ random variables. The value of *n* is *0, 1, 2* …, then $\left\{X_{n},n\in T\right\}$ is called a Markov chain. When supposing that the random process is $\left\{X_{n},n\in T\right\}$ ; if it satisfies any integer n∈T , any $j_{0},j_{1},\ldots ,j_{n+1}\in J$ , its state transition probability is (Ching and Ng, 2006):


(2) $$P\left\{\begin{array}{l} X_{n+1}=j_{n+1}|X_{0}=j_{0},\\ X_{1}=j_{1},\ldots .X_{n}=j_{n} \end{array}\right\}=P\left\{X_{n+1}=j_{n+1}|X_{n}=j_{n}\right\}$$

Subsequently, for the transition matrix *M* containing the absorbing state, the Markov chain whose initial state row vector is *S*, after *n* rounds of iterations, reaches its final state *S_final_* as:


$$SM^{n}=S_{\mathrm{final}}$$

where *n* is a sufficiently large positive integer, and the probability of a transition state (zone state) in *S_final_* is close to 0. In this study, the probability of the absorbing state represents the probability of creating a scoring opportunity (*TC*) and the probability of failing to create a scoring opportunity (*NTC*).

#### Calculating competitive relevance (performance relevance)

After calculating the probability of creating a scoring opportunity, a new state transition probability is calculated according to a deflection formula (Lames, 1991)


(3) $$\delta TP=C+B\times 4\times TP\left(1-TP\right)$$

where TP is the transition probability, δTP is the transition probability after the change, the constant C=1 , B=5 (Lames, 1991; Pfeiffer, 2005). In this process, every element (transition probability) in the initial matrix will be modified. To ensure that the sum of the rows of the modified matrix would still be 1, other cells in the same row in the matrix are calculated by using the compensation formula (Liu and Hohmann, 2013b)


(4) $$\delta TP_{yi}=-\left(TP_{yi}/\left(1-TP_{x}\right)\right)\times \delta TP_{x}$$

Afterward, the Markov chain model is used to calculate the probability of creating a scoring opportunity for the newly obtained matrix, and this new probability is compared with the result of the initial matrix calculation to determine the impact of changes between the states of the game (changes between the zones = passing) on the creation of scoring opportunities.

#### Data processing

First, the descriptive statistics of the passes between the different zones derived from the state transition matrix were calculated. The chi-square test was used to compare the general passing data of the top three teams in the 2018 World Cup against different opponents (*n*_possession *vs.* possession_ = 6; *n*_possession *vs.* direct_ = 8; *n*_direct *vs.* possession_ = 4; *n*_direct to direct_ = 3). Afterward, the Markov chain transition matrix was used to simulate and calculate the influence of the change of state in the game on the creation of scoring opportunities. The significance level was set to α=0.05 . According to Cohen (1988), the effect size is ${\mathrm{Cramer}}^{\prime }s\;\varphi \left(\mathrm{Phi}\right)$ , and the threshold is $\begin{array}{l} 0<\mathrm{Small}<0.1/\sqrt{k-1}<\mathrm{medium}<0.3/\sqrt{k-1}<\mathrm{large}\\ \;<0.5/\sqrt{k-1}<\mathrm{larger}<1.0/\sqrt{k-1} \end{array}$ ; in this study, the k value was 4. Data were processed by using SPSS (ver. 26, IBM, Chicago, United States) and Python (ver. 3.8), and graphical visualization was performed by using the online drawing tool draw.io (v. 14.8.4).

## Results

### Descriptive analysis of the passing paths of the top three teams in the 2018 world cup

**Figure 3:**
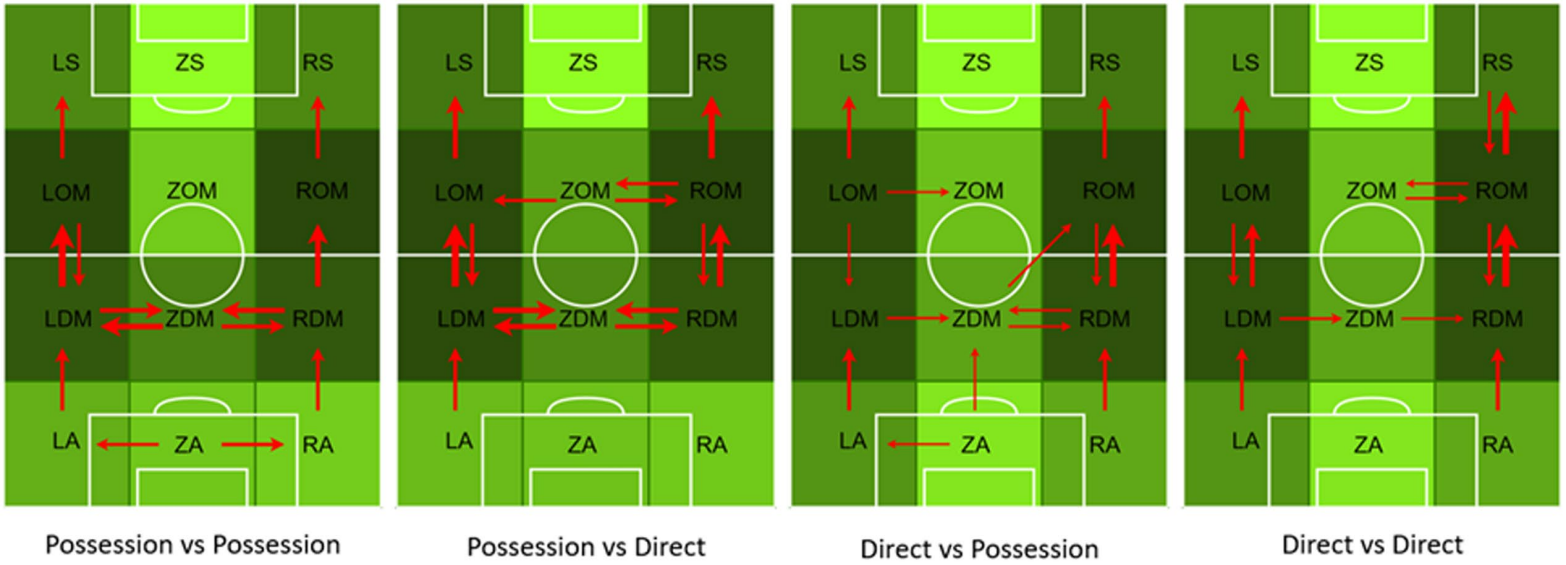
Descriptive analysis of actions zones and passing paths among top three teams in the World Cup 2018.

Figure 3 shows the descriptive analysis of the passing paths of the top three teams in the 2018 World Cup. The depth of the color of the field zones represents the frequency of technical and tactical activities in the area, and the thickness of the arrow represents the closeness of the connection between the areas. The top three teams rarely passed the ball across regions (with only a few long passes being observed), and all of the teams focused on using the wing area to organize their offenses. However, the differences in the passing and active areas of the different playing style teams were significant (Possession play vs. direct play, $\chi^{2}=437.871,{\mathrm{Cramer}}^{\prime }s\;\varphi =0.179,p<0.001$ ). Moreover, Croatian and Belgium teams, which exhibit possession playing styles, tended to use their own midfield zones (LDM, ZDM, and RDM) to organize their offenses, whereas France, which is a direct-play style team, tended to directly form an offense through their own backside zones (LA and RA). Furthermore, the differences in the passing and active areas of the same style of play teams against opponents of different styles were also significant (possession play vs. different opponents, $\chi^{2}=210.606,{\mathrm{Cramer}}^{\prime }s\;\varphi =0.152,p<0.001$ ; direct play vs. different opponents, $\chi^{2}=180.596,{\mathrm{Cramer}}^{\prime }s\;\varphi =0.200,p=0.007$ ).

### Diagnostic analysis of the passing paths of the top three teams in the 2018 World Cup

**Figure 4:**
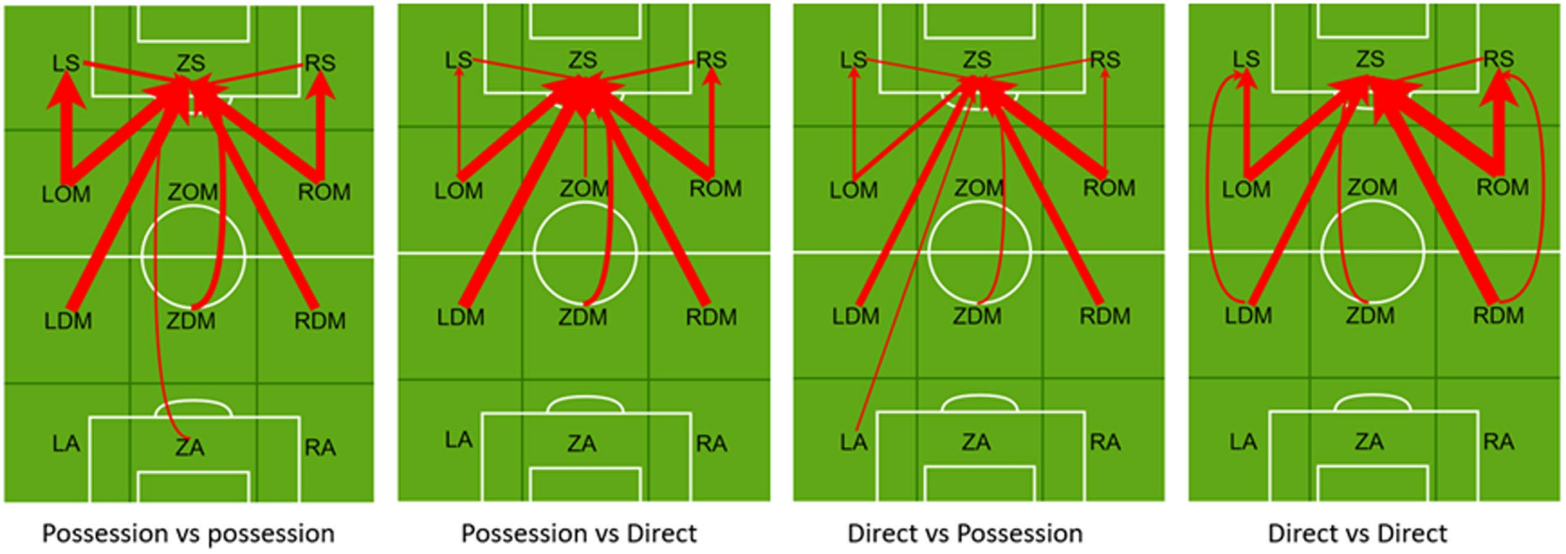
Diagnostical analysis of passing routines among top three teams in the World Cup 2018.

Table 3; Figure 4 illustrate the diagnostic analysis of the passing paths of the top three teams in the 2018 World Cup. For the top three teams, regardless of whether Croatia and Belgium teams focused on possession, or France focused on direct play, the diagnostic analysis observed that cross-regional passes (long passes) to the forward center (ZS) and to the side of the opponent’s half (LS and RS) have a positive effect on the creation of scoring opportunities. Especially for the direct playing style of France, when facing the same direct playing opponent, increasing side-way attack can increase its chance of scoring.

**Table 3:** Diagnostical analysis of passing routines among top three teams in the World Cup 2018.

Rank	Possession						Direct					
	vs. Possession			vs. Direct			vs. Possession			vs. Direct		
	From	To	PR	From	To	PR	From	To	PR	From	To	PR
1	LOM	ZS	0.490%	ROM	ZS	0.449%	ROM	ZS	0.425%	ROM	ZS	0.545%
2	ROM	ZS	0.447%	LDM	ZS	0.449%	RDM	ZS	0.387%	RDM	ZS	0.441%
3	LDM	ZS	0.443%	LOM	ZS	0.415%	LDM	ZS	0.380%	ROM	RS	0.428%
4	LOM	LS	0.423%	RDM	ZS	0.402%	LOM	ZS	0.353%	LOM	ZS	0.422%
5	RDM	ZS	0.407%	ZDM	ZS	0.306%	LOM	LS	0.253%	LDM	ZS	0.379%
6	ROM	RS	0.373%	ROM	RS	0.304%	ROM	RS	0.216%	LOM	LS	0.328%
7	ZDM	ZS	0.343%	RS	ZS	0.279%	ZDM	ZS	0.213%	RDM	RS	0.190%
8	LS	ZS	0.308%	LOM	LS	0.236%	LS	ZS	0.207%	ZDM	ZS	0.177%
9	RS	ZS	0.294%	LS	ZS	0.235%	RS	ZS	0.201%	RS	ZS	0.176%
10	ZA	ZS	0.261%	ZOM	ZS	0.233%	LA	ZS	0.200%	LDM	LS	0.175%

PR, Performance relevance.

## Discussion

The aim of this study was to analyze the paths and covering zones in the offensive sequence of high-level football games. The results found that (1) the top three teams in the 2018 World Cup tended to form offenses in the wing area, and possession-play teams (Croatia and Belgium) had more passes in their own midfielder zones than direct-play teams (France); and (2) cross-regional forward passes, especially passes toward the forward middle zone, had a particularly important positive impact on creating scoring opportunities.

From the offensive mode of the top three teams in the 2018 World Cup, the flanker areas are still effective offensive areas, which is also consistent with the studies on previous international football tournaments (Xue et al., 2015; Yamada and Hayashi, 2015; Mitrotasios et al., 2022). As attacks through middle zones will encounter greater defensive resistance, the side-way attack is a more effective offensive method (Grehaigne et al., 2002; Diana et al., 2017), which can disrupt the opponent’s defensive balance and increase the probability of a successful offense (Fernandez-Navarro et al., 2016). However, this study found that teams with different playing styles used the field areas differently. The possession playing Croatia and Belgium exhibited more lateral passes in the midfield than direct playing France, whereas France made more use of side areas on its own backcourt. For the top three teams in the 2018 World Cup, the difference in the use of covering areas and paths may be due to teams of different playing styles actively adopting their own style of play. Evidence indicates that stronger teams dominated ball possession against their opponents and shows that more stable patterns of play independently of the evolving score-line (Lago, 2009; Lago-Peñas and Dellal, 2010). Previous studies also have shown that both FC Barcelona and Manchester United (in the 2011 UEFA Champions League Finale) were possession-play teams (Sarmento et al., 2014a), although Manchester United was not as strong as FC Barcelona in that match, the players still had been trying and insisting on passing and controlling in the midfield area in the Finale (Liu and Hohmann, 2013a). During a football game, the team with the higher ball possession rate tends to be stronger (Hughes and Franks, 2005); therefore, they are also able to maintain possession of the ball in the midfield area (Casal et al., 2017) and have a higher possession rate in the opponent’s half and the 35-m area of the frontcourt (the attacking 1/3; this does not consider match status). In addition, the center of the formation (the centroid) in the game will also move forward accordingly (Clemente et al., 2013). In contrast, direct-play (counterattack) teams have a relatively low ball possession rate in the game, especially in the midfield. Therefore, direct playing teams are more inclined to use the wing to organize and launch offenses, especially from the sidelines of their own backcourt. This finding is also consistent with the research of Yi et al. (2019).

This study also found that for teams with both styles of play, passing from the side toward the forward center of the field (the ZS area) can better help in creating scoring opportunities. Therefore, the possession playing style teams can appropriately increase the cross-area long pass with the target of the front middle. Research on the European Cup and World Cup has shown that teams do not easily change the playing style in the game (Casal et al., 2017); however, teams that flexibly combine possession play and direct play in different situations can achieve better results (Yi et al., 2019). These findings also support the results of our study. During a football game, researchers have long known that the middle area of the frontcourt is an important area that is used to create scoring opportunities (Worthington, 1975; Hughes, 1990; Xue et al., 2015); thus, the early research of Pollard and Reep (1997) proposed that the ball should be introduced into this area as soon as possible to form a shot. This theory has considerably affected England and Norway teams (Larson, 2001). An early study (Grehaigne et al., 2002) showed that the area most likely to be intercepted and counterattacked by the opponent in the offense is the opponent’s middle area of the backcourt (ZOM, for the attacking team, the middle of the forecourt); however, the opponent’s penalty area (which is located in the frontcourt for the attacking team) is not the area where the defenders are most likely to steal the ball and counterattack (Grehaigne et al., 2002). Therefore, after combining the data of Bate (1988) and Dufour (1993), the target area of a cross-area long pass (the counterattack) should be the area between the goal area and the penalty area (around the penalty point). However, it should be noted that the abovementioned studies were conducted several decades ago. With the development of football, high position press and formation forward pressure tactics have gradually become a trend. Therefore, the target area of the pass in a quick counterattack and a positional attack is also different. The target area of a long pass for a quick counterattack and a positional attack should be combined with the area where the opponent’s formation is located; generally, the target is the area between the opponent’s central defender and the goalkeeper (Larson, 2001), which likely represents the attacking team’s middle front. Therefore, in a quick counterattack, the target area may be slightly far from the defender’s penalty area, whereas in a positional attack, the target area may be closer to the defender’s goal area.

Furthermore, pattern analysis is always an interesting topic in sports, which provides coaches and players with important information and helps to win the match. Previous studies also introduced pattern detection using T-pattern (Pic Aguilar, 2017) or Polar coordinates (Castañer et al., 2016; Maneiro Dios and Amatria Jiménez, 2018) or mixed method (Castañer et al., 2017) which are already sophisticated methods (Magnusson, 2019). Comparing with these studies, the current study used Markov Chain transition matrix model to describe and diagnose zone patterns of World Cup top teams, combining with style of play, it offers a new perspective to solve such problems. A difference between current method and T-pattern is that Markov Chain transition matrix method is based on event-sequence and not temporal sensitive for the temporal distances between events. It is worth noting in the future study to add some temporal factors.

The limitation of this study was that only the data of three teams were used. The current sample size between teams of two playing styles is not balanced enough and may not fully reflect the general situation of the two styles of play, but the characteristics of the top teams in the 2018 World Cup were still representative and showed the latest development trend of playing style and attacking pattern in football games. Through this study, coaches can understand different styles of play and development trends of football, as well as combine the data from this study with other studies, to make proper tactical responses in future games. In future research, situational factors (such as different playing styles, team strengths, match statuses, and match time) can be incorporated to analyze the changes in team play.

## Conclusion

This study analyzed and diagnosed the offensive patterns of the top three teams in the 2018 World Cup based on the Markov chain transition matrix. By constructing a transfer matrix, we demonstrated the methods that the top teams with different playing styles used in their offenses. Both playing style teams mainly used the wing area in their offense. Furthermore, the possession playing team organized more passes in the midfield, whereas the direct-play teams made more use of the side zones of their backcourt. In addition, the combination of possession play and counterattack was a new trend that emerged in the 2018 World Cup.

Although the possession playing style has prevailed in the past decade, the success of the French national team in the 2018 World Cup and the failure of Germany and Spain (who were in pursuit of pass and control tactics) have caused researchers to re-examine the game style. The efficiency of offense in the game has become increasingly important. Furthermore, possession-play football and direct play football are not incompatible, and offensive efficiency and scoring are ways to achieve game success.

## Data availability statement

The raw data supporting the conclusions of this article will be made available by the authors, without undue reservation.

## Ethics statement

Ethical review and approval was not required for the study on human participants in accordance with the local legislation and institutional requirements. Written informed consent for participation was not required for this study in accordance with the national legislation and the institutional requirements.

## Author contributions

TL conceptualized the study and wrote the original draft preparation. TL and CZ contributed to the methodology. XS, LZ, and CZ contributed to data collection and visualization. TL, JZ, and LY reviewed and edited the manuscript. All authors contributed to the article and approved the submitted version.

## Funding

This study was supported by the National Social Science Fund of China (18CTY011).

## Conflict of interest

The authors declare that the research was conducted in the absence of any commercial or financial relationships that could be construed as a potential conflict of interest.

## Publisher’s note

All claims expressed in this article are solely those of the authors and do not necessarily represent those of their affiliated organizations, or those of the publisher, the editors and the reviewers. Any product that may be evaluated in this article, or claim that may be made by its manufacturer, is not guaranteed or endorsed by the publisher.
